# Surface Coating with Hyaluronic Acid-Gelatin-Crosslinked Hydrogel on Gelatin-Conjugated Poly(dimethylsiloxane) for Implantable Medical Device-Induced Fibrosis

**DOI:** 10.3390/pharmaceutics13020269

**Published:** 2021-02-17

**Authors:** Haejin Joo, Jonghyun Park, Chanutchamon Sutthiwanjampa, Hankoo Kim, Taehui Bae, Wooseob Kim, Jinhwa Choi, Mikyung Kim, Shinhyuk Kang, Hansoo Park

**Affiliations:** 1 Department of Integrative Engineering, Chung-Ang University, Seoul 06974, Korea; 9h_03_j3@naver.com (H.J.); ssong.scha@gmail.com (C.S.); 2 Department of Plastic and Reconstructive Surgery, Chung-Ang University Hospital, Seoul 06973, Korea; davidparke@caumc.or.kr (J.P.); hkkiim@cau.ac.kr (H.K.); psbth@cau.ac.kr (T.B.); kimws@cau.ac.kr (W.K.); 3 Department of Radiation Oncology, Chung-Ang University Hospital, Seoul 06973, Korea; bonnybee@caumc.or.kr; 4 Department of Pathology, Chung-Ang University Hospital, Seoul 06973, Korea; mkkim@cau.ac.kr

**Keywords:** polydimethylsiloxane, surface coating, hydrogel, Schiff-base reaction, hydrophilicity, biocompatibility

## Abstract

Polydimethylsiloxane (PDMS) is a biocompatible polymer that has been applied in many fields. However, the surface hydrophobicity of PDMS can limit successful implementation, and this must be reduced by surface modification to improve biocompatibility. In this study, we modified the PDMS surface with a hydrogel and investigated the effect of this on hydrophilicity, bacterial adhesion, cell viability, immune response, and biocompatibility of PDMS. Hydrogels were created from hyaluronic acid and gelatin using a Schiff-base reaction. The PDMS surface and hydrogel were characterized using nuclear magnetic resonance, X-ray photoelectron spectroscopy, attenuated total reflection Fourier-transform infrared spectroscopy, and scanning electron microscopy. The hydrophilicity of the surface was confirmed via a decrease in the water contact angle. Bacterial anti-adhesion was demonstrated for *Pseudomonas aeruginosa, Ralstonia pickettii,* and *Staphylococcus epidermidis*, and viability and improved distribution of human-derived adipose stem cells were also confirmed. Decreased capsular tissue responses were observed in vivo with looser collagen distribution and reduced cytokine expression on the hydrogel-coated surface. Hydrogel coating on treated PDMS is a promising method to improve the surface hydrophilicity and biocompatibility for surface modification of biomedical applications.

## 1. Introduction

Polydimethylsiloxane (PDMS) is a biocompatible silicone polymer. PDMS possesses very attractive physical and chemical properties such as low toxicity, biocompatibility, optical transparency, elastomeric properties, gas permeability, ease of fabrication, and low manufacturing costs [1]. These desirable properties are of value in biomedical applications of medical devices such as silicone implants, contact lenses, catheters, microfluidic devices, coatings, and lithography [2,3,4]. Despite these advantages, the hydrophobic surface of PDMS can have side effects such as adsorbing nonspecific proteins and hydrophobic analytes, enabling bacterial adhesion, and limiting the flow of aqueous fluids [5]. Consequently, when PDMS is inserted into a blood-contact environment, health problems arise, and poor performance can be a concern for the long-term applicability of microfluidics [6]. Therefore, the improvement of the surface wettability of PDMS is very important for biomedical applications.

Recently, various surface modifications for improving hydrophilicity and antifouling have been studied [7,8,9,10]. A simple and fast method to create a hydrophilic PDMS surface is to expose the surface directly to oxygen plasma to create silanol (Si–OH) groups on the surface. However, the hydrophobic properties of PDMS can rapidly recover, and hence hydrophilicity is difficult to sustain [11,12,13]. Thus, surface modifications of PDMS using biomaterials that improve the hydrophilicity and inhibit bacterial growth have been investigated. The efficient and easy-to-implement “grafting-to” method is very popular for the surface modification of silicon-based biomaterials to achieve tunable surface properties [14]. In particular, the use of a self-assembled monolayer such as (3-aminopropyl)triethoxysilane (APTES) stabilizes the surface modification. Additionally, APTES treatment creates a strong intermediate connection between the PDMS surface and extracellular matrix (ECM) protein, which produces a cell microenvironment on the surface, leading to enhanced biocompatibility and improved cell behavior [15]. Gelatin is an adhesive protein that stabilizes the hydrogel coating on the PDMS surface [16,17]. Gelatin conjugates on the surface of PDMS via reaction with 1-ethyl-3-(3-dimethylaminopropyl)carbodiimide (EDC)/N-hydroxysuccinimide (NHS) for immobilization [18,19,20].

Hydrogels are three-dimensional polymers with excellent swelling properties and similarities with soft tissue, such as hydrophilicity and high affinity for water. Moreover, the many advantages of hydrogels, including good biocompatibility, tunable mechanical properties, and biodegradability, are attractive for biomedical applications [21]. Specifically, a crosslinked hydrogel comprising hyaluronic acid (HA) and gelatin is attracting great attention in biomedical applications such as tissue engineering [22,23]. HA and gelatin are derived from ECM and are widely used for hydrogels [24]. HA is a hydrophilic polymer with biological properties, including angiogenesis, modulation of inflammation, and controlled and tunable cell response [25,26,27]. Gelatin derived from collagen is a low immunogenic polymer that allows biodegradation and cell adhesion; thus, it has been used in tissue engineering and cell culture [28,29]. Therefore, optimization of the gelatin/HA ratio will achieve faster and denser angiogenesis and affect the stiffness of the hydrogel. Based on these previous studies, the rate of proteolysis, as well as the ratio and spatial distribution of gelatin to HA to improve angiogenesis, have been optimized [30]. HA and gelatin are crosslinked by a Schiff-base reaction to improve their mechanical and biological properties. Schiff-base crosslinks have been widely used to prepare hydrogels because of their rapid gelation and excellent biocompatibility [31]. Modified HA and gelatin have been used in a Schiff-base reaction to link aldehyde and amine groups at neutral pH [32,33].

**Scheme 1:**
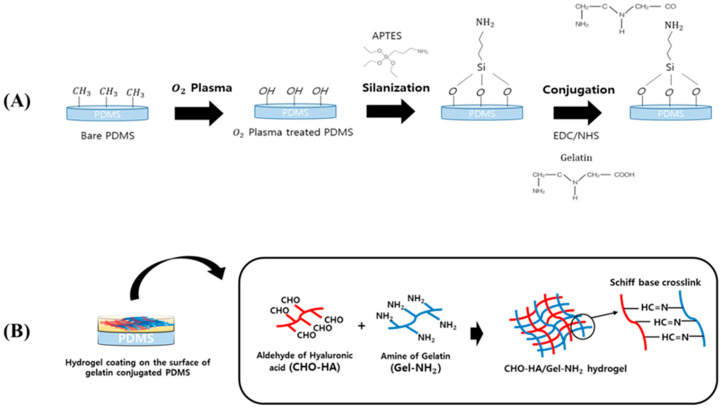
Illustration of the poly(dimethylsiloxane) (PDMS) surface treatment: (**A**) Gelatin conjugation with PDMS using (3-aminopropyl)triethoxysilane (APTES); (**B**) Hydrogel coating on the surface of gelatin-conjugated PDMS using 1-ethyl-3-(3-dimethylaminopropyl)carbodiimide (EDC)/N-hydroxysuccinimide (NHS) reaction.

In this study, we modified the PDMS surface using a hydrogel of crosslinking HA and gelatin to improve hydrophilicity and biocompatibility (Scheme 1). Then, the modified surface was analyzed using various methods of surface characterization, including nuclear magnetic resonance (NMR), X-ray photoelectron spectroscopy (XPS), attenuated total reflection Fourier-transform infrared spectroscopy (ATR-FTIR), and scanning electron microscopy (SEM). In addition, the hydrophilicity of the surface was analyzed via the water contact angle. Bacterial anti-adhesion was evaluated using *Pseudomonas aeruginosa*, *Ralstonia pickettii*, and *Staphylococcus epidermidis*. Viability of human-derived adipose stem cells (hASCs) was determined by the LIVE/DEAD assay. Furthermore, capsule tissue responses were analyzed in vivo 8 weeks after implant insertion.

## 2. Materials and Methods

### 2.1. Materials

PDMS elastomer (Sylgard^®^ 184 silicone elastomer kit) was purchased from Dow Corning, Midland, Michigan, USA. NHS (assay ≥ 98%, molecular weight (MW): 115.09 g/mol), APTES (assay ≥ 99%, MW: 221.37 g/mol, density: 0.946 g/mL at 25 °C (lit.)), Gelatin powder (from porcine skin, type A, 175 blooms) and EDC were purchased from Sigma-Aldrich, Seoul, South Korea. Hyaluronate (medium MW = 1,000,000−1,600,000 Da) was purchased from SK bioland, Cheongju, South Korea. Sodium periodate (NaIO4; ACS reagent; ≥99.8%), ethylenediamine Reagent Plus^®^ (≥99%), and anhydrous ethylene glycol (≥99.8%) were obtained from Sigma-Aldrich, Seoul, South Korea. HyClone phosphate-buffered saline (PBS) was purchased from Fisher Scientific, Waltham, MA, USA. Spectra/Por^®^ 3 dialysis membrane (molecular weight cut off (MWCO): 3500 Da) and Spectra/Por^®^ 4 dialysis membrane (MWCO: 12,000−14,000 Da) were obtained from Fisher Scientific, Waltham, Massachusetts, USA. Dulbecco’s modified Eagle’s medium (DMEM), Dulbecco’s PBS (DPBS/Modified 1×, pH 7.4), trypsin 0.25% (1×) solution, fetal bovine serum, and antibiotic/antimycotic solution (100×) were purchased from GE Healthcare Life Sciences HyClone Laboratories, Logan, UT, USA. hASCs were isolated from adipose tissue obtained undergoing lipid suction. Cellulose membranes (MWCO: 3,500 Da) were purchased from HANKOOK SPECTRUM, Seoul, South Korea. Distilled water (DW) was used for the preparation of solutions and washing. *S. epidermidis* (ATCC 35984; Manassas, VA, USA), *P. aeruginosa* (ATCC 27853), and *R. pickettii* (ATCC 27511) were obtained from the Korean Agricultural Culture Collection, Jeonju, South Korea. A LIVE/DEAD BacLight bacterial viability kit was purchased from Thermo Fisher Scientific (Waltham, MA, USA). All chemicals were used without further purification.

### 2.2. Preparation of Gelatin-Conjugated PDMS Surfaces

#### 2.2.1. Preparation of PDMS

The Sylgard^®^ 184 silicone elastomer kit is the precursor of the PDMS solution. This comprises a base (pre-polymer) and curing agent (crosslinker). For PDMS device fabrication, the base and curing agent were mixed in a 10:1 weight ratio. This mixture was transferred to a petri dish and placed in a vacuum desiccator for 5 min to remove air bubbles. After curing in an oven at 70 °C for 1 h, 0.2 cm thick PDMS blocks were produced using a 1.2 cm diameter spherical puncher. The PDMS surface was washed with 70% ethanol and dried for 12 h in a fume hood.

#### 2.2.2. Surface Conjugation of PDMS with Gelatin

The cleaned PDMS surface was treated with oxygen plasma using O_2_ plasma equipment (CUTE-1B, Femto Science, Hwaseong, South Korea) to remove the methyl group from the surface and attach the hydroxyl group. The power supply was fixed at 100 W and the O_2_ flow rate was 20 sccm at a pressure of 5 × 10^−2^ Torr for 1 min. Next, this was dipped into DW containing 5 wt% APTES (Sigma-Aldrich, St Louis, MO, USA) with constant stirring (200 rpm) at 60 °C for 2 h. After APTES treatment for silanization, the PDMS surfaces were washed twice with 70% ethanol to remove unreacted APTES molecules with PDMS. After washing with PBS one more time, a gelatin-conjugation solution was separately prepared in 100 mL of PBS by adding appropriate amounts of EDC (0.958 g, 50 mmol) and NHS (0.575 g, 50 mmol) to the prepared PBS solution. Gelatin powder (5 g from porcine skin, type A, 175 blooms) was added to the solution, and the PDMS surfaces were immersed in the gelatin reaction mixture. Then, gelatin was conjugated to the surface with constant stirring at 200 rpm for 2 h at 55 °C (on a stirring hotplate of 130–135 °C). Unreacted gelatin and EDC/NHS reaction residue were thoroughly washed twice with 70% ethanol. The gelatin-conjugated PDMS surfaces were dried in a fume hood for 12 h.

### 2.3. Hydrogel Coating on Gelatin-Conjugated PDMS Surfaces

#### 2.3.1. Preparation of Oxidized HA

To prepare the hydrogel for the Schiff-base reaction, HA and gelatin were modified. First, HA was oxidized by sodium periodate (NaIO_4_). HA (1 g) was dissolved in 100 mL of DW, and 0.547 g of sodium periodate with 5 mL of DW was added to the HA solution with constant stirring at room temperature in the dark for 24 h. Next, ethylene glycol was added and reacted for 1 h to stop the previous reaction. The obtained solution was thoroughly dialyzed for three days using dialysis tubing (MWCO: 12,000–14,000 Da, Spectra/Por membrane) in deionized water (DI) and lyophilized to obtain oxidized HA (CHO–HA).

#### 2.3.2. Preparation of Gelatin Amine

Gelatin powder (5 g) (from porcine skin, type A, 175 blooms) was dissolved in 100 mL of DW, and 15.8 mL of ethylenediamine was added to the solution and allowed to react for 5 min. The pH was adjusted to 5 with 5 M hydrochloric acid, and a 2.68 g of EDC was added. The reaction mixture was stirred at 37 °C for 18 h, and then dialyzed (MWCO: 3500 Da, Spectra/Por membrane) in DI for 48 h to remove excess ethylenediamine and EDC. The dialyzed solution was lyophilized at −80 °C to obtain a gelatin product with more amine functional groups.

#### 2.3.3. Surface Coating with Hydrogel on Gelatin-Conjugated PDMS Surfaces

The hydrogel was made from modified HA and gelatin through a Schiff-base reaction. The aldehyde of CHO–HA and the amine of gelatin were cross-linked in the CHO–HA solution and gelatin amine (Gel–NH_2_) solution at a specific concentration. The obtained hydrogel was coated onto the gelatin-conjugated PDMS surface with high binding stability between the hydrogel and PDMS surface. The surface was treated on one side only, and the hydrogel was coated onto this treated area. To prepare a suitable hydrogel for PDMS surface coating, lyophilized CHO–HA, Gel–NH_2_, and PBS were mixed to produce 20% concentration solutions. Subsequently, these solutions were mixed to fabricate hydrogels with ratios of 7:3, 5:5, and 3:7 of CHO–HA/Gel–NH_2_ on the surface of the gelatin-conjugated PDMS.

### 2.4. Characterization and Instrumentation

#### 2.4.1. Polydimethylsiloxane PDMS Modified with Gelatin

Before coating, ATR-FTIR (Nicolet 6700, Thermo Scientific, Waltham, MA, USA) and XPS were used to characterize the surface of PDMS conjugated with gelatin. Each spectrum was estimated in the range of 400 to 4000 cm^−1^, with an average of 200 scans at room temperature. Elemental analysis of the gelatin-conjugated PDMS surface was performed using XPS (K-alpha+, Thermo Fisher Scientific, Waltham, MA, USA) equipped with a spot area (on a 90° sample) of 400 µm and a monochromatic A1 Kα X-ray source (200.0 eV). Each sample was cut into a circle with 1.2 cm diameter, and C1s, O1s, Si2p, and N1s were examined.

#### 2.4.2. Hyaluronic Acid and Gelatin Modified for Schiff-Base Crosslink

^1^H-NMR and ATR-FTIR were used to evaluate the modified HA and gelatin. ^1^H-NMR spectra were acquired at room temperature with a Varian 600 NMR spectrometer using a solvent of D_2_O. The sample solution (0.5 mL) was transferred to a 5 mm NMR tube. Tetramethylsilane was used as an internal reference. Subsequent analysis was performed using the MestReNova program. ATR-FTIR analysis was performed to confirm the expected pendant functionalities. Each spectrum was estimated in the range of 400 to 4000 cm^−1^, with an average of 200 scans at room temperature.

#### 2.4.3. Scanning Electron Microscopy (SEM)

SEM (S-3400N, Hitachi, Tokyo, Japan) was used to measure the morphology of the gelatin-conjugated PDMS compared to that of untreated bare PDMS at 10 kV acceleration voltages. In addition, the binding of the hydrogel on the gelatin-conjugated PDMS surface and that on the untreated bare PDMS were compared. Samples were mounted onto a stub and coated with platinum layers via ion sputter coating for 120 s (E-1010, Hitachi, Tokyo, Japan).

#### 2.4.4. Water Contact Angle

A water-contact-angle goniometer (Phoenix 300, S.E.O. Co., Ltd., Ansung, Korea) was used to evaluate the hydrophilicity on the surface of the gelatin-conjugated PDMS and compare this to that of the untreated bare PDMS. In addition, hydrogel coating on gelatin-conjugated PDMS was measured by varying the hydrogel ratio (CHO–HA/Gel–NH_2_). A droplet of water was dropped onto the sample, and the contact angle was measured.

#### 2.4.5. Swelling Ability and Degradation

The swelling ability and degradation of the hydrogels bonded with the gelatin-conjugated surface of PDMS were evaluated by weight. CHO–HA/Gel–NH_2_ hydrogels coated onto the gelatin-conjugated PDMS were immersed in a PBS solution at pH 7.4 and incubated at 37 °C in orbital shaker at 150 rpm. The swollen samples were removed from the PBS after 1, 2, 3, 4, 7, 14, 26, 38, 52, and 125 h. The samples were wiped off with tissue paper, weighed, and placed back into the PBS solution. The swelling ratio was calculated using the following equation:

(1) $$\mathrm{Swelling}\;\mathrm{ratio}\;=\;\frac{\mathrm{WS}-\mathrm{WD}}{\mathrm{WD}}\;\times \;100\;(\%),\;$$

where WS and WD are the weights of the swollen and dry samples, respectively.

### 2.5. Bacterial Anti-Adhesion

*S. epidermidis* (ATCC 35984; Manassas, VA, USA), *P. aeruginosa* (ATCC 27853), and *R. pickettii* (ATCC 27511) were used to evaluate the bacterial anti-adhesion effect on the surface of the hydrogel-coated PDMS compared to that of the bare PDMS. All samples were sterilized with ethylene oxide gas for 12 h and stored in a fume hood for 8 h. Then, the gelatin-conjugated PDMS was coated with hydrogels at ratios of 7:3, 5:5, and 3:7 of CHO–HA/Gel–NH_2_, which had been filtered using a 0.4 µm filter tube. Bacterial cell suspensions of *R. pickettii* and *S. epidermidis* were prepared in nutrient media and a *P. aeruginosa* suspension was prepared in Luria–Bertani media. Bare PDMS and hydrogel-coated PDMS were immersed in 20 mL of LB medium and incubated in a shaking incubator at 150 rpm and 37 °C for 12 h. After incubation, the samples were washed twice with DPBS (HyClone) at pH 7.4, and bacterial adhesion and viability on the samples were confirmed using the LIVE/DEAD™ BacLight™ Bacterial Viability Kit (Thermo Fisher, Waltham, MA, USA). Bacteria were stained with a mixture of SYTO 9 and propidium iodide in an incubator for 15 min and measured via fluorescence microscopy (Olympus CKX-41, Tokyo, Japan). The semi-quantitative results are summarized in a bar chart using ImageJ (1.53 g, Wayne Rasband, NIH, Bethesda, MD, USA). The intensity was calculated using the following equation:

(2) $$\mathrm{Fluorescence}\;\mathrm{intensity}\;=\;\frac{\mathrm{bacteria}\;\mathrm{area}\;\left(\mathrm{LIVE}/\mathrm{DEAD}\right)}{\mathrm{Total}\;\mathrm{area}}\;\times \;100\;(\%)$$

### 2.6. Cell Viability

hASCs were cultured in low-glucose DMEM supplemented with 1% antibiotic/antimycotic solution (100×) and 10% fetal bovine serum. Media were changed once every two days, and cultured cells were harvested using trypsin 0.25% (1×) solution. The obtained cells were seeded at a concentration of 2 × 10^4^ cells/mL on the surfaces of the bare PDMS and the hydrogel-coated, gelatin-conjugated PDMS (CHO–HA/Gel–NH_2_ at 7:3, 5:5, and 3:7) in a 24 well plate. Then, all plates were incubated in an incubator at 37 °C with 5% CO_2_ for 24, 48, and 72 h. The viability of hASCs on the surfaces of bare PDMS and hydrogel-coated, gelatin-conjugated PDMS was measured using a LIVE/DEAD viability/cytotoxicity kit for mammalian cells (Thermo Fisher Scientific) after 24, 48, and 72 h. Cells were stained using a mixture of 4 mM calcein AM and 2 mM ethidium homodimer, which are LIVE/DEAD kit solutions in PBS, and incubated in an incubator at 37 °C with 5% CO_2_ for 30 min.

### 2.7. In Vivo Experiments

#### 2.7.1. Animals

Twelve five-week-old male Sprague Dawley rats weighing 220–250 g were used for the in vivo experiments. The procedures were performed in the Animal Research Laboratory of Chung-Ang University and approved by the Animal Review Committee of Chung-Ang University. An intraperitoneal injection of 50 mg/kg of tiletamine/zolazepam and 5 mg/kg of xylazine hydrochloride was used to anesthetize the rats. An approximately 2 cm incision was made in the dorsal area via aseptic techniques. A subpanniculus carnosus layer pouch was created on both sides through the dorsal incision line, and four separate pockets were formed at the 2, 4, 8, and 10 o’clock positions. For rat experimental studies, round silicone block implants (PDMS) of 1.2 × 1.2 × 0.2 cm were made as explained above. Hydrogel-coated PDMS (CHO–HA/Gel–NH_2_ at 7:3, 5:5, and 3:7) and bare PDMS were inserted into separate pockets created in the subcarnosus layer of 12 rats. Four weeks after the operation, radiation exposure was performed on all rats to exaggerate fibrous capsule formation. Irradiation was conducted with a 6 eV electron beam at a dose rate of 300 cGy/min. The rats were locally irradiated on their backs with a single dose of 15 Gy. A 1 cm bolus was applied to control the depth of radiation. Rats were sacrificed at eight weeks postoperation, and capsule tissue was harvested for evaluation. Evaluations and comparisons were performed in a double-blind manner. Additionally, statistical analysis was performed using the Mann–Whitney test.

#### 2.7.2. Histology and Immunohistochemistry

Tissue sections from harvested samples were stained with hematoxylin and eosin and Masson’s trichrome. Capsular tissue thickness was measured in hematoxylin and eosin stain slides, and collagen distribution was confirmed using Masson’s trichrome stain slides.

#### 2.7.3. Quantitative Polymerase Chain Reaction (qPCR)

Extraction and purification of total ribonucleic acid (RNA) from harvested rat tissue was performed using Trizol^®^ reagent. (Invitrogen, Carlsbad, CA, USA). The extracted and purified RNA was replicated via PCR using RTPreMix (Bioneer, Daejeon, Korea). RT-qPCR was conducted with Power SYBR Green PCR Master Mix (Life Technologies, Carlsbad, CA, USA), using the StepOnePlus Real-Time PCR system (AB Applied, Life Technologies). All gene expression data were normalized to data from the housekeeping gene *Gapdh*. The primer sequences are listed in Table 1.

## 3. Results and Discussion

### 3.1. H-NMR Spectra of HA and Gelatin

**Figure 1:**
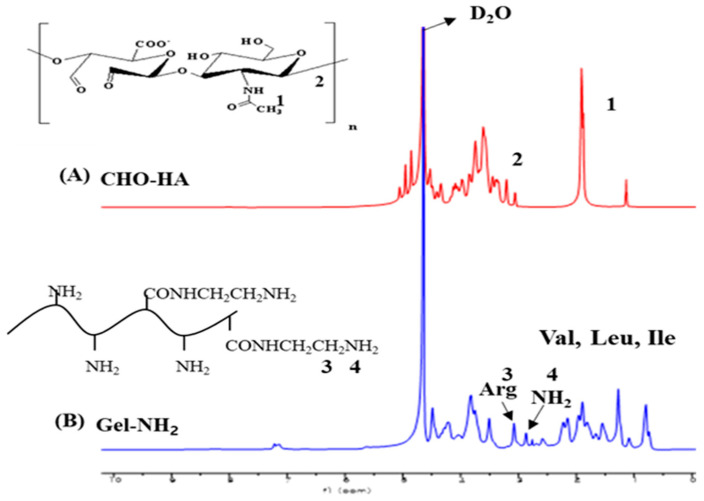
^1^H- nuclear magnetic resonance (NMR) results of modified hyaluronic acid (HA) and gelatin. (**A**) CHO–HA: oxidized HA, (**B**) Gel–NH_2_: gelatin amine, Val: valine, Leu: leucine, Ile: isoleucine, Arg: arginine.

The ^1^H-NMR results (Figure 1) were used to confirm the chemical structures after dialysis and freeze-drying of the CHO–HA and Gel–NH_2_ samples. Figure 1A shows the CHO–HA spectra, where ^1^H-NMR resonant signals can be observed at 1.9 ppm (acetamide protons, NH–CO–CH_3_), 3.0–3.9 ppm (proton of sugar unit, CH–O and CH_2_–O), 4.3 and 4.4 ppm (anomeric protons, OCH–O), and 5.0–5.1 ppm (hemiacetal proton-formed aldehyde and adjacent hydroxyl groups) [34]. Meanwhile, the gel–NH_2_ spectra are presented in Figure 1B, where the resonant signals in the 0.8–1.5 ppm range indicate the amino acids valine, leucine, and isoleucine. The signal in the 1.5–3.0 ppm range is due to the aliphatic carbon protons of amino acids such as arginine, leucine, lysine, proline, glycine, and asparagine. The peaks at 3.2–4.3 ppm represent the resonance signals of the α–CH of the amino acids [35]. The structure of gelatin is rich in glycine, proline, and hydroxyproline [36].

### 3.2. Surface Characterization of PDMS Conjugated with Gelatin

#### 3.2.1. ATR-FTIR Spectra of PDMS Surface

**Figure 2:**
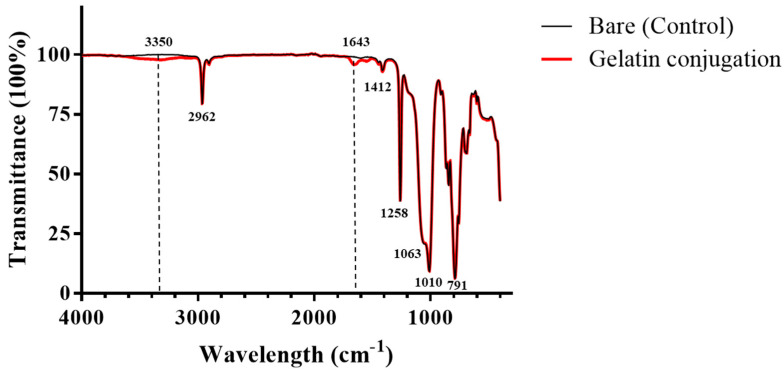
Attenuated total reflection Fourier-transform infrared spectroscopy (ATR-FTIR) results for bare poly(dimethylsiloxane) (PDMS) and gelatin-conjugated PDMS surfaces.

The PDMS surface was modified by gelatin conjugation via a chemical method. The ATR-FTIR results (Figure 2) confirm the successful gelatin-conjugation of PDMS compared to the untreated PDMS. All spectra contain peaks at 791 cm^−1^, which is characteristic of Si–(CH_3_)_2_, and 1010 and 1063 cm^−1^, which indicate the Si–O–Si stretching vibrations, while the peak at 1258 cm^−1^ is due to various C–H vibrations of the methyl group from the PDMS surface. In addition, the peak at 2962 cm^−1^ is characteristic of methyl CH [37]. Both spectra appear similar. However, in the spectrum of the gelatin-conjugated PDMS, the peak at 3350 cm^−1^ indicates N–H stretching and O–H stretching that were observed at 3700–3000 cm^−1^, whereas that at 1643 cm^−1^ appeared due to the C=O stretching of the carboxyl group [38,39]. These peaks show that gelatin was successfully conjugated with PDMS.

#### 3.2.2. XPS Results of PDMS Surface

**Figure 3:**
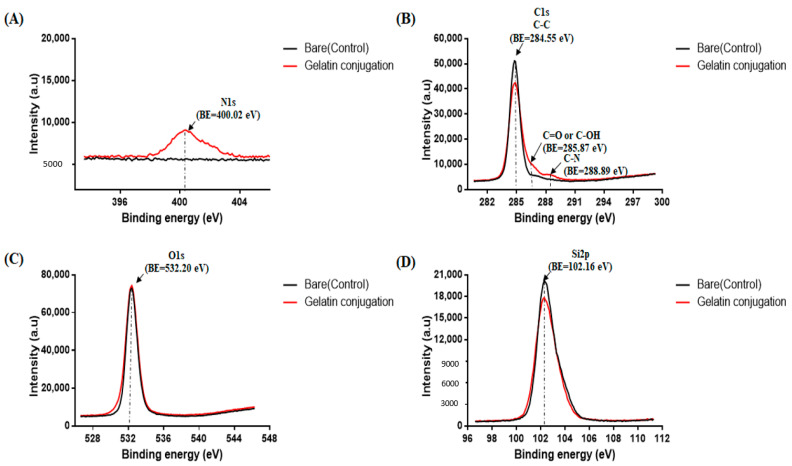
X-ray photoelectron spectroscopy (XPS) results of gelatin-conjugated poly(dimethylsiloxane) (PDMS) surface compared to those of bare PDMS. BE: binding energy. XPS elemental composition analyses in the regions of (**A**) N 1s (Nitrogen), (**B**) C 1s (Carbon), (**C**) O 1s (Oxygen) and (**D**) Si 2p (Silicon) on bare and gelatin conjugated PDMS.

The XPS results (Figure 3) confirmed the gelatin-conjugation of PDMS compared to bare PDMS. The binding energy range was selected to detect nitrogen, carbon, silicon, and oxygen. In particular, the location of the nitrogen signal is important as this indicates successful immobilization and amide bonding of the silane initiator and the PDMS surface. The gelatin-conjugated PDMS surface exhibited nitrogen peaks at the N1s binding-energy sites of 400.04 and 401.40 eV, which belong to the nitrogen of the primary amine. The bare PDMS surface showed a carbon peak (284.55 eV) at the C1s binding energy related to methylene (=CH_2_) or methyl (–CH_3_) groups. In addition, the gelatin-conjugated PDMS surface had a carbon peak (285.87 eV) at the carboxyl (C=O of –CHO) group and a single peak (288.89 eV) at the binding energy of the C-N group. These results show that the XPS spectra of both O1s and Si2p are consistent with the previously presented data. Furthermore, the results suggest that gelatin conjugation with PDMS successfully modified the PDMS surface via chemical methods.

### 3.3. Characterization and In Vitro Test of Surface Coating with Hydrogel

#### 3.3.1. Morphology

**Figure 4:**
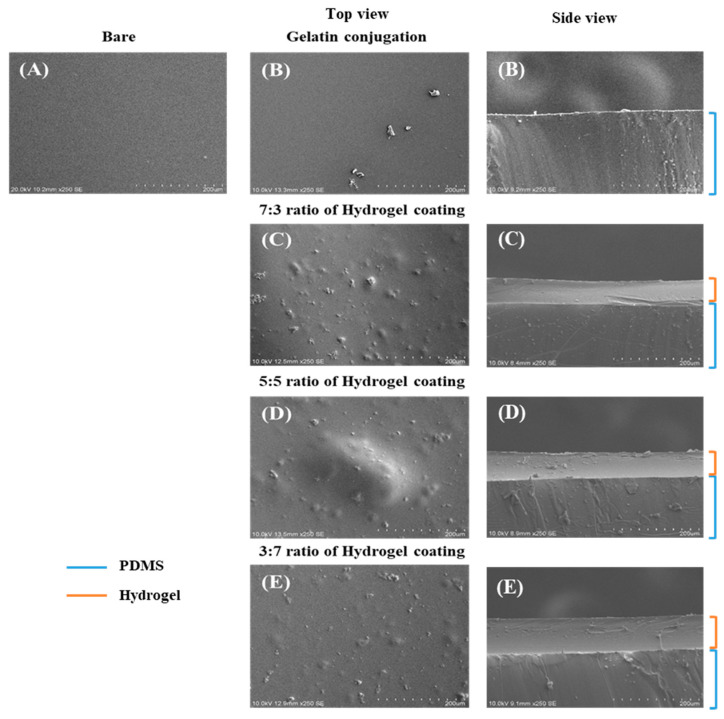
Top- and side-view scanning electron microscopy (SEM) images of the modified poly(dimethylsiloxane) (PDMS) surface compared to the bare PDMS surface. (**A**) Bare PDMS; (**B**) Gelatin-conjugated PDMS and hydrogel-coated groups with CHO–HA/Gel–NH2 of (**C**) 7:3, (**D**) 5:5, and (**E**) 3:7.

The SEM results (Figure 4) indicated successful gelatin conjugation with PDMS and the stability of the hydrogel with the modified PDMS surface compared to the surface of bare PDMS, showing that the various ratios of hydrogels were well bonded to the surface of the modified PDMS. These results confirmed that the binding ability of the hydrogel with the modified PDMS was stronger than that of bare PDMS. The surface of the gelatin-conjugated PDMS was uneven and irregular, while that of the bare PDMS was clean and smooth. After hydrogel coating on the gelatin-conjugated PDMS, the surface was coarse and rough. The side view (Figure 4) indicates that the hydrogel coating was uniform on the surface of the gelatin-conjugated PDMS. Furthermore, these results confirm that the hydrogel was successfully conjugated with PDMS, and the gelatin used to connect the hydrogel and the PDMS surface performed well.

#### 3.3.2. Water Contact Angle

**Figure 5:**
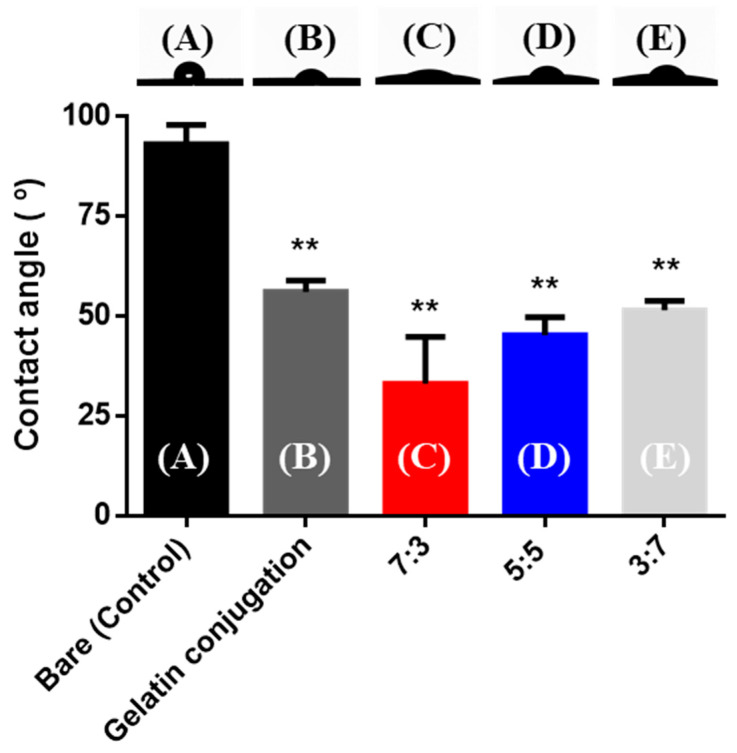
Contact-angle images and values of the modified poly(dimethylsiloxane) (PDMS) surface compared to the bare PDMS surface. (**A**) Bare PDMS; (**B**) Gelatin-conjugated PDMS and hydrogel coated groups with CHO–HA/Gel–NH_2_ (**C**) 7:3, (**D**) 5:5, and (**E**) 3:7. (** *p* < 0.01, Mann–Whitney test, compared to bare PDMS.)

The lower the water contact angle (θ, °), the higher the hydrophilicity of the surface. The water contact-angle results (Figure 5) show the hydrophilicity of the surface. After surface conjugation with gelatin, the contact angle decreased more than that of bare PDMS. Therefore, the modified PDMS surface was more hydrophilic than the bare PDMS surface. In addition, the contact angle values of the hydrogel coated onto the modified surfaces of the PDMS were lower than those of the bare PDMS. The contact angle values of the control, gelatin-conjugated, and CHO–HA/Gel–NH_2_ = 7:3, 5:5, and 3:7 groups were 93.15 ± 4.84, 56.22 ± 2.85, 33.14 ± 11.76, 45.25 ± 4.53, and 51.52 ± 2.45°, respectively. These results indicated that gelatin was conjugated to the surface of PDMS, and the hydrogel coating on the PDMS produced hydrophilic surfaces [40,41]. When comparing the contact angles of the hydrogels with CHO–HA/Gel–NH_2_ at ratios of 7:3, 5:5, and 3:7, the 7:3 ratio had the lowest value and hence the most hydrophilic surface. Therefore, HA is more hydrophilic than gelatin.

#### 3.3.3. Swelling Ability and Degradation

**Figure 6:**
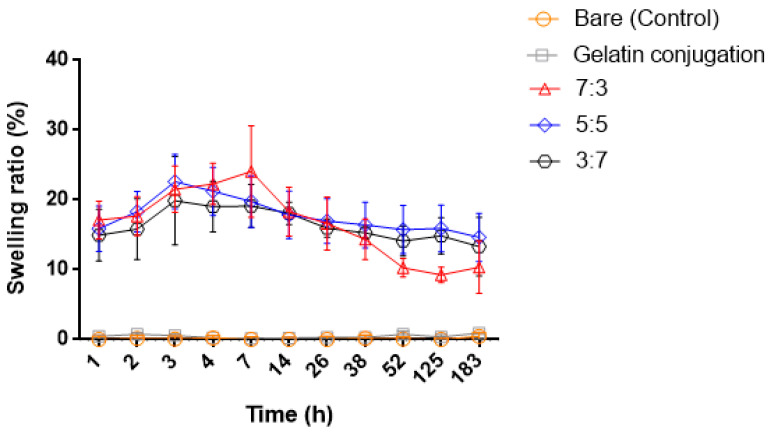
Comparison of swelling and degradation ability between bare, gelatin-conjugated poly(dimethylsiloxane) (PDMS), and PDMS coated with hydrogels with CHO–HA/Gel–NH_2_ ratios of 7:3, 5:5, and 3:7 over time.

At intervals of 1, 2, 3, 4, 7, 14, 26, 38, 52, 125, and 183 h, the swelling ability and degradation were evaluated over 7 days, as shown in Figure 6. In addition, the stability of the hydrogel-modified PDMS surface was compared to that of bare PDMS and gelatin-conjugated PDMS. When the hydrogel was bonded to the bare PDMS surface and immersed in PBS, the hydrogel immediately fell off the surface. The hydrogel bound on the gelatin-conjugated surface in PBS was maintained. For different ratios of CHO–HA/Gel–NH_2_ hydrogel-coated PDMS for the selected interval times, the results show the trends in swelling ability and degradation in PBS at 37 °C in a shaking incubator at 150 rpm. There was no swelling ability with either bare PDMS or gelatin-conjugated PDMS. However, compared to these, the hydrogel-coated PDMS groups exhibited swelling ability. In the case of the 7:3 hydrogel group, the swelling ratio increased, and then rapidly decomposed after 7 h compared to that of the other groups. In the 5:5 and 3:7 groups, the swelling ratio increased and gradually decreased after 3 h. The swelling ability is related to the degree of hydrophilicity. Depending on the gelatin content, the stiffness of the hydrogel is determined. When the proportion of gelatin was higher than that of hyaluronic acid, the hydrogel had a higher stiffness. The higher the ratio of gelatin, the lower the hydrophilicity and swelling ratio [42]. The ratio of 3:7 (CHO–HA/Gel–NH_2_) is the stiffest hydrogel with the lowest hydrophilicity and longest stability. The ratio of 7:3 hydrogel decomposed faster, related to hydrophilicity (Figure 6). According to the results, hydrogels containing more HA degraded faster than gelatin. The contact-angle results (Figure 5) showed that the 7:3 hydrogel had the highest hydrophilicity.

#### 3.3.4. Bacterial Anti-Adhesion

**Figure 7:**
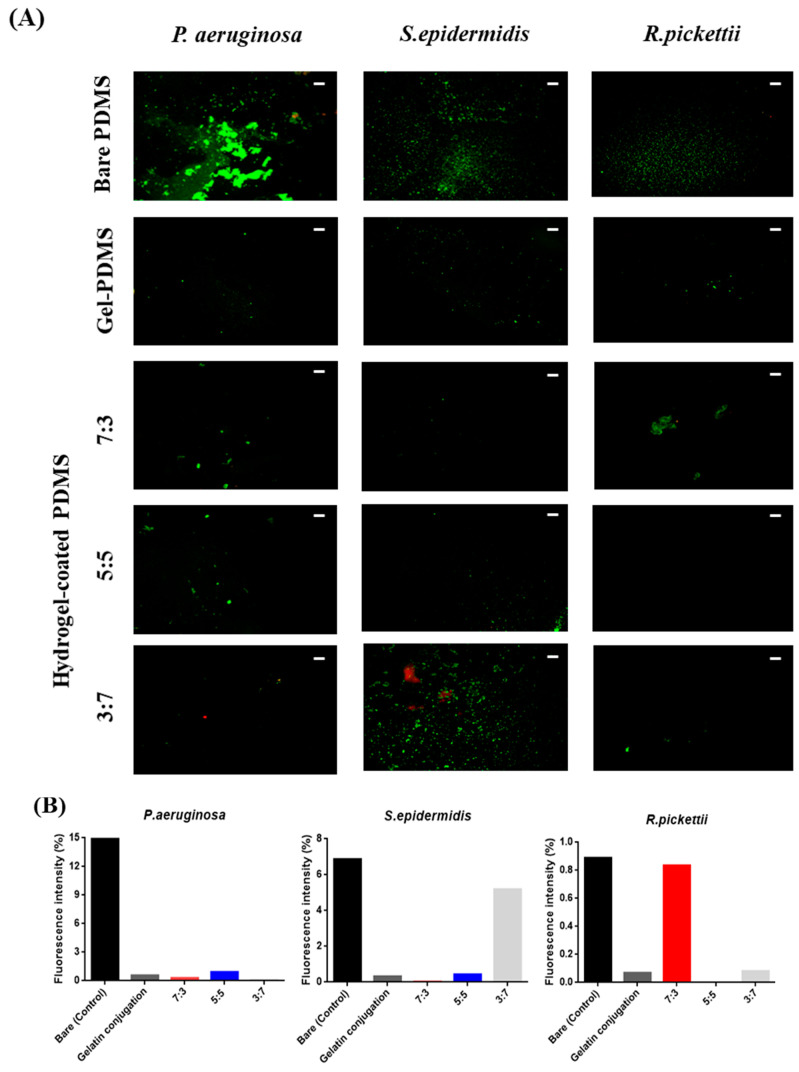
(**A**) Bacterial anti-adhesion effect on *P. aeruginosa*, *S. epidermis*, and *R. pickettii* on the surfaces of bare poly(dimethylsiloxane) PDMS and PDMS modified with gelatin and hydrogel composed of CHO–HA/Gel–NH_2_ (the scale bar represents 100 µm); (**B**) Semi-quantitative results of bacteria LIVE/DEAD images.

We evaluated the bacterial anti-adhesion effect on the surface of the hydrogel-coated, gelatin-conjugated PDMS and gelatin-conjugated PDMS against *S. epidermidis*, *P. aeruginosa*, and *R. pickettii* using LIVE/DEAD staining assays. The distributions of live and dead bacteria on the surface of the bare PDMS, gelatin-conjugated PDMS, and hydrogel-coated PDMS after immersion in the bacterial suspension are shown in Figure 7. In the case of *P. aeruginosa*, all surfaces of the hydrogel-coated PDMS and gelatin-conjugated PDMS showed decreased adhesion compared to that of bare PDMS; among these, the 3:7 hydrogel exhibited the lowest adhesion. With *S. epidermidis*, all treated PDMS exhibited a reduction in bacterial adhesion compared to that of bare PDMS. Among the hydrogel groups, the 3:7 hydrogel demonstrated the highest adhesion related to the stiffness of the hydrogel [43]. As the stiffness of the hydrogel increased, the degree of bacterial adsorption increased in the distribution of *S. epidermidis*. Finally, bacterial adhesion of *R. pickettii* on the hydrogel-coated PDMS and gelatin-conjugated PDMS surfaces was reduced compared to that of the untreated PDMS surface. Therefore, bacterial anti-adhesion was reduced in most of the hydrogel-coated PDMS and gelatin-conjugated PDMS samples compared to bare PDMS.

#### 3.3.5. In-Vitro Cell Viability

**Figure 8:**
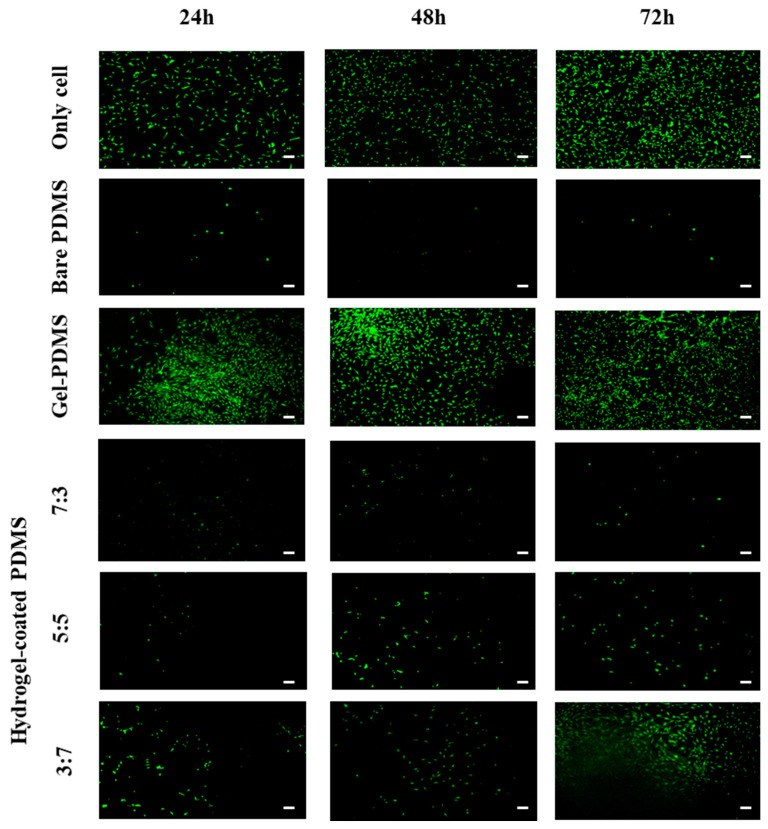
Cell viability on the surfaces of bare poly(dimethylsiloxane) PDMS and PDMS modified with gelatin and hydrogel composed of CHO–HA/Gel–NH_2_. (The scale bar represents 100 µm.).

The viability of hASCs was evaluated via fluorescence microscopy using the LIVE/DEAD assay. Figure 8 shows hASCs cultured on the surface of the hydrogel-coated PDMS and gelatin-conjugated PDMS. The viability of hASCs was higher on the surface of the gelatin-conjugated PDMS than that on the bare PDMS surface. In addition, among the hydrogel-coated PDMS surfaces, the results showed that the hASC viability was the highest in the PDMS coated with the 3:7 hydrogel, whereas cell viability was the lowest in the PDMS coated with the 7:3 hydrogel. These results can be attributed to the higher proportion of gelatin being related to enhanced cell adhesion and proliferation [44]. In addition, HA-based biomaterials have been reported to inhibit cell adhesion because of their hydrophilicity, and the cells tend to selectively adhere to neutral, hydrophobic, or multi-cation surfaces of the material [45,46].

### 3.4. In Vivo Analysis of Tissue Evaluation and Quantification

#### 3.4.1. Capsular Thickness

**Figure 9:**
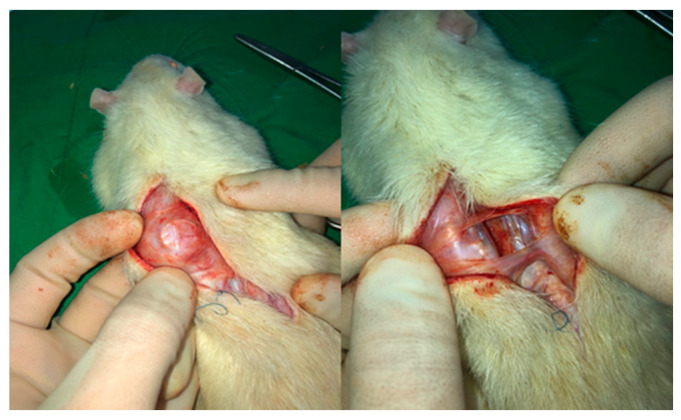
Rats sacrificed after eight weeks showing capsule formation around inserted samples.

**Figure 10:**
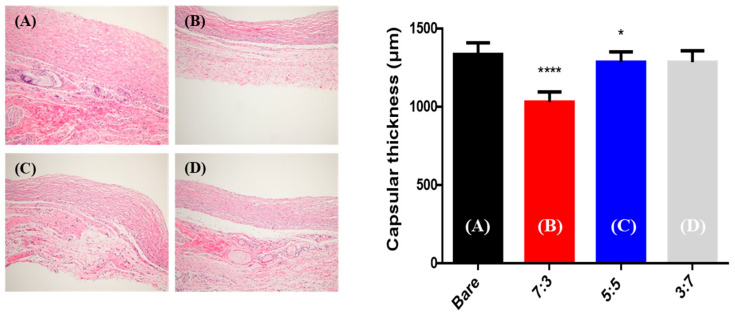
Histologic images of capsular tissue and bar graphs indicating thickness of capsules eight weeks after operation (hematoxylin and eosin staining, 200×). (**A**) Bare (control), (**B**) 7:3, (**C**) 5:5, and (**D**) 3:7 groups. (NS: not significant, * *p* < 0.05, **** *p* < 0.0001, Mann–Whitney test, compared to bare poly(dimethylsiloxane) (PDMS).)

Twelve rats were euthanized eight weeks after insertion of the samples (Figure 9). The capsule thickness differed according to the degree of capsular contracture [47]. Figure 10 presents the histologic images and bar graphs showing the thicknesses of the capsules. The thickness of capsular tissues in the control, 7:3, 5:5, and 3:7 groups was 1335 ± 75, 1030 ± 66, 1285 ± 67, and 1285 ± 75 µm, respectively. The capsular thickness of the hydrogel-coated PDMS was less than that of the bare PDMS. In particular, the capsular thickness of the 7:3 hydrogel-coated PDMS decreased more than those of the other hydrogel-coated PDMS samples.

#### 3.4.2. Collagen Arrangement and Collagen Types I and III

**Figure 11:**
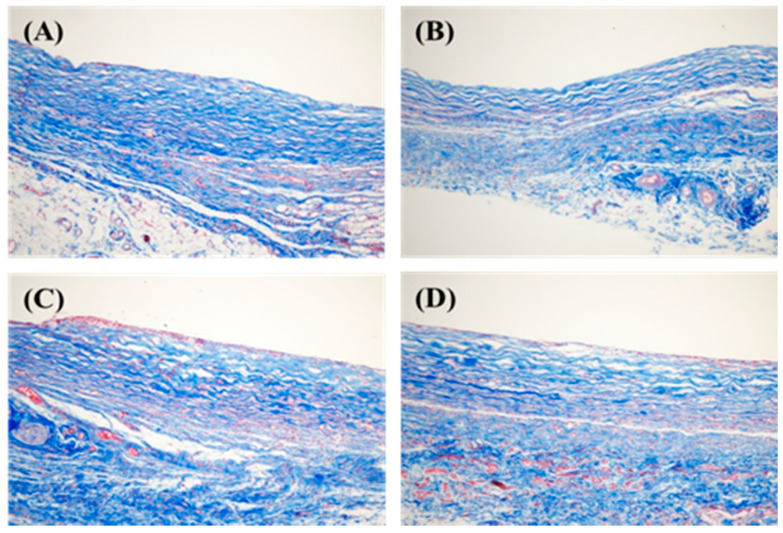
Arrangement of collagen fiber stained with Masson’s trichrome (200× magnification). (**A**) Bare (control), (**B**) 7:3, (**C**) 5:5, and (**D**) 3:7 groups.

**Figure 12:**
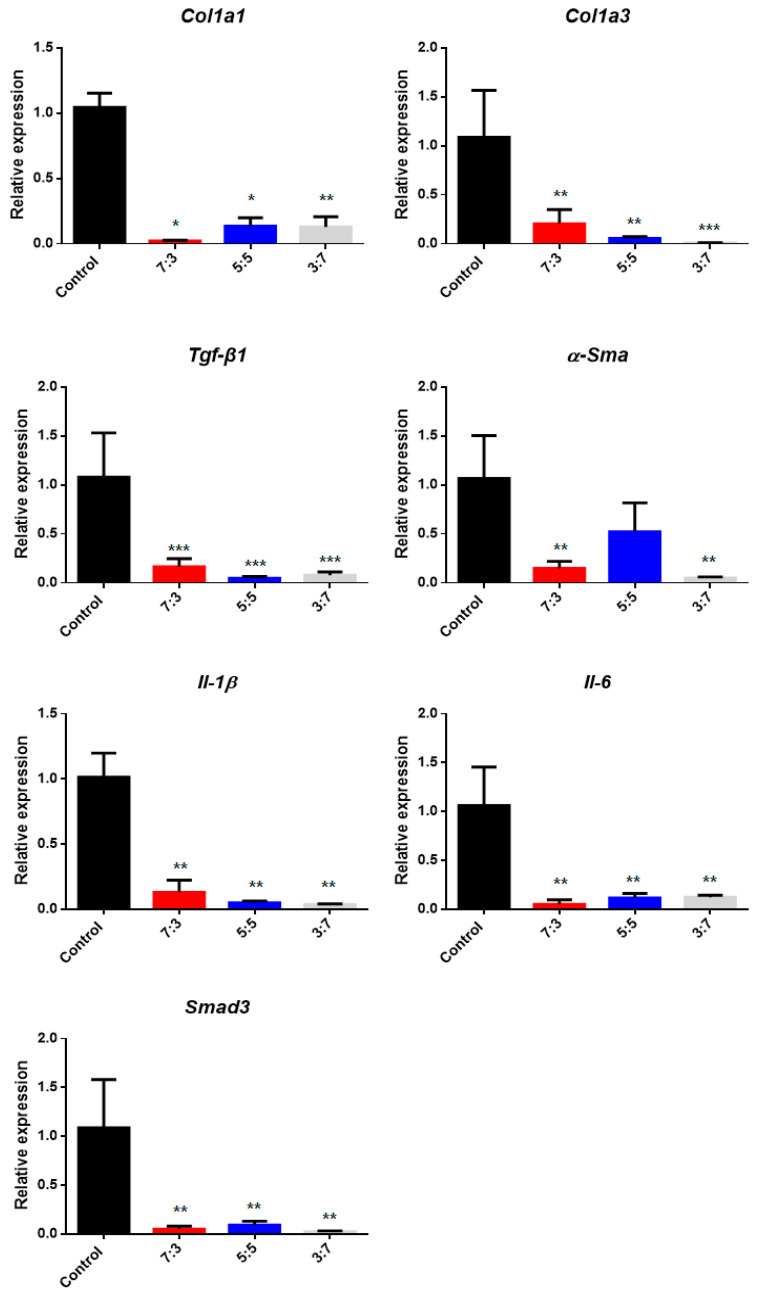
Relative expression of genes in tissue for hydrogel-coated poly(dimethylsiloxane) PDMS (CHO–HA/Gel–NH_2_ = 7:3, 5:5, and 3:7) compared to the control group (bare PDMS). (* *p* < 0.05, ** *p* < 0.01, *** *p* < 0.001, Mann–Whitney test, compared to bare PDMS).

To detect collagen fiber distribution in the capsular tissue, Masson’s trichrome staining was performed, as shown in Figure 11. The collagen fibers of the hydrogel-coated PDMS were relatively thin and more loosely distributed than those of the bare PDMS. Figure 12 shows that the relative messenger RNA (mRNA) expression of collagen type I and collagen type III in the hydrogel-coated, gelatin-conjugated PDMS groups were significantly lower than those in the bare PDMS groups. The relative mRNA ratios of collagen type I in the control, 7:3, 5:5, and 3:7 groups were 1.0447 ± 0.0352, 0.0178 ± 0.0076, 0.1358 ± 0.0637, and 0.1286 ± 0.0786, respectively. Meanwhile, the relative mRNA expression levels of collagen type III in the control, 7:3, 5:5, and 3:7 groups were 1.0867 ± 0.4799, 0.2021 ± 0.1470, 0.0535 ± 0.0196, and 0.0071 ± 0.0030, respectively. More specifically, the hydrogel coating affected collagen alignment and expression of collagen types I and III.

3.4.3. qPCR

A qPCR can monitor amplification products in real time to measure target gene expression. The presence of target genes causes an inflammatory response that stimulates the immune response by signaling foreign substances via T cells. The relative mRNA expression of target genes (Table 1) is related to immune responses, such as capsular contracture and fibrosis [48,49]. The results (Figure 12 and Table 2) from the analysis confirm that expression all the target genes in the tissue on the surface of the hydrogel-coated modified PDMS was lower than that in tissue with bare PDMS.

## 4. Conclusions

PDMS is a widely used material in medicine. Various types of implantable silicone devices that are inserted into the body are in great demand, especially as breast implants, and are currently used for esthetic and reconstruction purposes. Breast implants are manufactured by treating cohesive silicone gel with multiple layers of PDMS shell. In our study, the PDMS elastomer is a material used for patients in clinical surgery. This study was designed with a material similar to that of the implant shell used in clinical practice for future clinical application. As a limitation, this PDMS shell was not filled with cohesive gel like a breast implant, and it was not possible to determine whether the shell leaked, but we confirmed with electron microscopy that there was no problem such as surface cracking. After inserting of general implants such as catheters and breast implants, side effects such as bacterial adhesion, nonspecific protein adsorption, and inflammation may occur. However, the hydrogel coating with gelatin-conjugated PDMS conferred antibacterial adhesion properties, reduced capsular thickness, and decreased relative expression of cytokines for fibrosis. However, this coating has a disadvantage in that the hydrogel may fail when physical force is applied; therefore, this must be carefully handled during surgery.

The control group presented in this study could be considered a traditional PDMS material. In the present study, PDMS surfaces were conjugated with gelatin to enhance hydrophilicity for the binding ability of the hydrogel. Characterization of the modified surface via ATR-FTIR, XPS, SEM, and water contact-angle measurements confirmed the presence of gelatin on the modified surface. The hydrogel coated onto the surface of the gelatin-conjugated PDMS had reduced hydrophobicity and increased biocompatibility compared to these characteristics in bare PDMS. The water contact-angle value was reduced by 40% in the gelatin conjugation group and by 64% in the 7:3 group compared to that in the conventional PDMS material. Hydrogel coating was found to decrease bacterial adhesion on the surface compared to that in bare PDMS. A degree of connective tissue occurs physiologically around a foreign body; however, progression to a thick, hard capsule causes local inflammation, dislocation, and implant deformation. The histologic results of the capsule thickness were non-significant compared to the expression of collagen-related genes. Capsular thickness decreased by 23% in the 7:3 group compared to that in the control group. Although the experimental groups had relatively thinner peri-implant capsule formation, the gene expression studies showed significant differences, and therefore the results seem inconsistent. This could be due to several reasons, such as the small sample size of the study and the duration of the study. Although this study was designed to create an adequate amount of capsule formation around the implant for comparison, including radiation therapy, capsular tissue formation was not as high as expected in all groups. In fact, the harvested capsular tissue was grossly thin in all groups. Additional weeks could improve capsular thickness and increase the difference between the experimental and control groups. Moreover, radiation exposure promptly after implant insertion, instead of at 4 weeks postoperation, could have exaggerated capsular formation more by interfering with the wound healing process. We have confirmed that our surface modification method using hydrogels has an effect on preventing harmful immune responses such as fibrosis and capsule formation in an in vivo rat model. Compared to other methods to fabricate biocompatible surfaces of PDMS, it is possible to create implants that can deliver drugs to specific areas using the drug-delivery function of hydrogels, as well as lowering the original hydrophobicity and causing antibacterial effects and inflammatory reactions. Therefore, this biocompatible surface-modification method for hydrogel coating with gelatin-conjugated PDMS can be used for implantable biomedical devices such as drug-deliverable silicone implants.

## Figures and Tables

**Table 1:** Rat primer sequences used for RT-qPCR amplifications.

Gene	Direction	Primer Sequence	Reference
*Gapdh*	Forward	5′-GGCACAGTCAAGGCTGAGAATG-3′	NM_017008.3 *
	Reverse	5′-ATGGTGGTGAAGACGCCAGTA-3′	
*Col1a1*	Forward	5′-GACATGTTCAGCTTTGTGGACCC-3′	NM_053304 *
	Reverse	5′-AGGGACCCTTAGGCCATTGTGTA-3′	
*Col1a3*	Forward	5′-TTTGGCACAGCAGTCCAATGTA-3′	NM_032085 *
	Reverse	5′-GACAGATCCCGAGTCGCAGA-3′	
*Tgf-β1*	Forward	5′-CACCGGAGAGCCCTGGATA-3′	NM_021578 *
	Reverse	5′-TCCAACCCAGGTCCTTCCTA-3′	
*α-Sma*	Forward	5′-ATCCTGACCCTGAAGTATCCGATA-3′	NM_031004 *
	Reverse	5′-CCACGCGAAGCTCGTTATAGA-3′	
*Smad3*	Forward	5′-CGCATGAGCTTCGTCAAAGG-3′	NM_013095.3 *
	Reverse	5′-CCGATCCCTTTACTCCCAGTG-3′	
*Il-1β*	Forward	5′-CACCTCTCAAGCAGAGCACAG-3′	M98820 *
	Reverse	5′-GGGTTCCATGGTGAAGTCAAC-3′	
*Il-6*	Forward	5′-TCCTACCCCAACTTCCAATGCTC-3′	E02522 *
	Reverse	5′-TTGGATGGTCTTGGTCCTTAGCC-3′	

* GenBank accession numbers

**Table 2:** Relative mRNA gene expression in PDMS coated with hydrogel.

Gene	Control	7:3	5:5	3:7
*Col1a1*	1.0447 ± 0.1090	0.0209 ± 0.0059 *	0.1358 ± 0.0637 *	0.1286 ± 0.0786 **
*Col1a3*	1.0867 ± 0.4799	0.2021 ± 0.1470 **	0.0535 ± 0.0196 **	0.0071 ± 0.0030 ***
*Tgf-β1*	1.0795 ± 0.4508	0.1636 ± 0.0848 ***	0.0481 ± 0.0153 ***	0.0780 ± 0.0331 ***
*α-Sma*	1.0660 ± 0.4364	0.1487 ± 0.0701 **	0.5214 ± 0.2950	0.0499 ± 0.0112 **
*Smad3*	1.0881 ± 0.4926	0.0499 ± 0.0301 **	0.0914 ± 0.0387 **	0.0240 ± 0.0085 **
*Il-1β*	1.0129 ± 0.1855	0.1294 ± 0.0941 **	0.0488 ± 0.0137 **	0.0358 ± 0.0050 **
*Il-6*	1.0622 ± 0.3944	0.0497 ± 0.0461 **	0.1135 ± 0.0471 **	0.1233 ± 0.0200 **

(NS: not significant, * *p* < 0.05, ** *p* < 0.01, *** *p* < 0.001 Mann–Whitney test, compared to control group).

## Data Availability

Data is contained within the article.
